# Structural insights into Cas9 mismatch: promising for development of high-fidelity Cas9 variants

**DOI:** 10.1038/s41392-022-01139-z

**Published:** 2022-08-04

**Authors:** Honghai Tang, Daqi Wang, Yilai Shu

**Affiliations:** 1 ENT Institute and Department of Otorhinolaryngology, Eye & ENT Hospital, State Key Laboratory of Medical Neurobiology and MOE Frontiers Center for Brain Science, Fudan University, Shanghai, China; 2 Institute of Biomedical Science, Fudan University, Shanghai, China; 3 NHC Key Laboratory of Hearing Medicine (Fudan University), Shanghai, China

**Keywords:** Genetic techniques, Structural biology

The recent research published in *Nature* by Bravo and colleagues has deeply clarified the mechanisms of Cas9 recognizing the base-pair mismatches by kinetics-guided Cryo-EM analysis, which provides a perspective for the development of next-generation Cas9 variants with high-fidelity.^1^

CRISPR-Cas9 is a type of endonuclease from bacteria and archaea, and can be programmed to target the genome under the guiding of a specific RNA. Although CRISPR-Cas9 has become one of the most popular tools for genome editing (e.g., the rapid generation of animal models, the steady-state performance optimization of plants, the treatment of hereditary diseases and oncology, etc.), the certain defects of Cas9 hinder its clinical application, especially off-target effects. To date, some strategies (e.g., reducing nonspecific DNA contacts, neutralizing the positive charge) can reduce the off-target effects of Cas9 due to mismatches,^2,3^ but the efficiency of Cas9 variants is also correspondingly decreased in the on-target DNA cleavage. Therefore, it is very urgent to explore the underlying mechanisms that Cas9 recognizes mismatches for developing next-generation Cas9 editing tools with high efficiency and high specificity.

Given that the activated intermediates before Cas9 cleavage are more suitable to help understand the mismatch mechanisms, it is necessary to capture a large amount of the pre-cleavage conformations of Cas9-sgRNA-DNA ternary complexes. To obtain the suitable structures of Cas9-sgRNA-DNA ternary complexes for elucidation of the off-target mechanisms of Cas9, the authors first used kinetic analysis to detect rates of Cas9 cleaving complementary strand (TS), which carries a contiguous 3-nt mismatch with sgRNA. In comparison to rapid on-target cleavage, the authors screened three substrates with 3-nt base mismatches from the PAM distal end to resolve the structures of Cas9-sgRNA-DNA ternary complexes. These substrates included the following DNA segments with triple mismatches 15–17 bp (15–17 mm), 12–14 bp (12–14 mm), and 18–20 bp (18–20 mm), the cleavage rates of which became higher in turn.^1^

To understand how Cas9 was activated in mismatched DNA substrates, the authors resolved two structures that Cas9 combined 12–14 mm DNA at different reaction time points. In structure after reaction for 5 min, the TS-sgRNA duplex of the PAM-distal end presented a linear conformation in comparison with the DNA–DNA duplex of the PAM-proximal end, and HNH domain (consists of two beta-sheets and four alpha-helices, which are similar to the structure of HNH endonuclease) was not observed; in the structure after reaction for 60 min, the TS-sgRNA duplex of the PAM distal end included both linear and kinked duplex conformations.^1^ This suggests that the linear duplex conformation resembles a transition state of Cas9 prior to HNH rearranging and docking to TS.^1^ Meanwhile, the structure displayed that the REC3 (recognition lobe 3) domain of Cas9 did not directly contact nucleotides from 12 to 14 in the TS-sgRNA, which was also supported by the structure of Cas9 combining 15–17 mm DNA, implying that the REC3 plays an important role in detecting PAM-distal mismatches. These data suggest that the linear conformation preceding the kinked conformation of the TS-sgRNA duplex is necessary for activation, and inhibition of the kinked duplex formation due to mismatches may avoid DNA cleavage by Cas9.^1^

To reveal how certain mismatches were recognized by Cas9 to obtain high-effective DNA cleavage in comparison with other mismatches, the authors determined the structure that Cas9 combined 18–20 mm DNA. Particles of the structure contained both linear and kinked duplex conformations, implying that this more tolerant mismatch also experiences a structural transition similar to that of 12–14 mm DNA.^1^ Meanwhile, HNH docking at the shearing site of the TS was observed in the structure.^1^ These indicate that Cas9 is the fully active conformation in the structure, and the kink of the TS-sgRNA duplex is concerned with HNH docking. In addition, in the structure, RuvC (comprises ten beta-sheets and nine alpha-helices, which share structural similarity with RNase H, for example, *Escherichia coli* RuvC) active site hydrolyzing nucleotide displayed a histidine-mediated catalytic model.^1^ The high-quality cryo-EM density of the helixes L1 and L2 was observed,^1^ the atomic interface interaction of which indicates that TS and NTS cleavage is couple. The 18–20 mm presented a distinctive duplex conformation at the position of the mismatch, which was stabilized by RuvC residues,^1^ implying that the residues only participated in the mismatch combining, instead of the on-target activation. Based on these structural works, the authors designed a high-fidelity variant (SuperFi-Cas9) with extreme-low mismatch rates and near wild-type cleavage efficiency, which has an ability to distinguish between on-target DNA and off-target DNA without harming the efficiency of DNA cleavage.^1^ But the efficacy of the variant still needs to be verified in cells or animal models in future works.

**Fig. 1:**
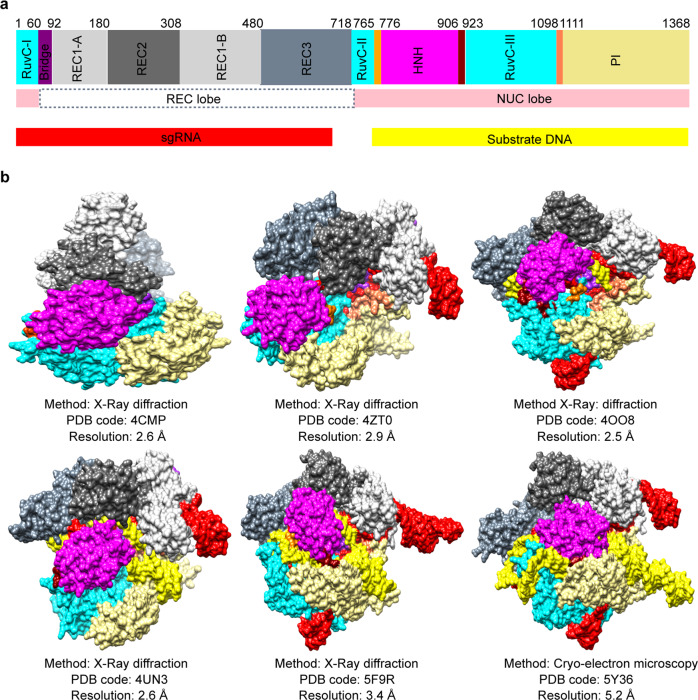
Structures of CRISPR-Cas9 at different states. **a** Domains of the Cas9-sgRNA-DNA ternary complex are marked by different colors. Cas9 contains 1368 amino acids. **b** The structures are determined by X-ray diffraction or Cryo-electron microscopy, including Cas9 monomer (PDB ID: 4CMP), Cas9-sgRNA binary complex (PDB ID: 4ZT0), and Cas9-sgRNA-DNA ternary complexes (PDB IDs: 4OO8, 4UN3, 5F9R, 5Y36)

In previous studies, many structural forms of CRISPR-Cas9 were determined, including Cas9 monomer (PDB ID: 4CMP), Cas9-sgRNA binary complex (PDB ID: 4ZT0), and Cas9-sgRNA-DNA ternary complexes (PDB IDs: 4OO8, 4UN3, 5F9R, 5Y36, and 6O0Y). In these structures, only the ternary complexes may be suitable for exploring the off-target effects of Cas9. However, their DNA substrates are only on-target DNA double strands without mismatch. And except 6O0Y,^4^ the residues Asp10 and His840 of the catalytic centers in the other structures are mutated into Ala,^5^ the atomic models of which are not the actually catalytic-active conformation (Fig. 1). For this, it is very difficult to clearly elucidate the mechanisms of Cas9 off-target effects in the active state using these structures. Therefore, the structures of Cas9-sgRNA–DNA ternary complexes in different mismatch states were resolved by Bravo et al. to compensate for the deficiencies of the earlier Cas9 structures, and have important implications for understanding and elucidation of the off-target mechanisms of Cas9. However, now it is not clear whether the rules revealed by Bravo et al. are appropriate for more complex forms of the off-target sites in the entire genome.

Taken together, Bravo and colleagues’ work provides a systematic study for Cas9 recognizing mismatches at the atomic level, and offers underlying structural insights into the off-target mechanisms of Cas9. Meanwhile, this work also provides structural guidance for the development of next-generation Cas9 variants with high efficiency and high specificity.
